# Presentation and evaluation of the teaching concept “ENHANCE” for basic sciences in medical education

**DOI:** 10.1371/journal.pone.0239928

**Published:** 2020-09-29

**Authors:** Karim Kouz, Sophie Eisenbarth, Alina Bergholz, Sonja Mohr

**Affiliations:** 1 Department of Anesthesiology, Center of Anesthesiology and Intensive Care Medicine, University Medical Center Hamburg-Eppendorf, Hamburg, Germany; 2 Department of Biochemistry and Molecular Cell Biology, University Medical Center Hamburg-Eppendorf, Hamburg, Germany; 3 Vice Deanery for Students’ Affairs, University Medical Center Hamburg-Eppendorf, Hamburg, Germany; Medical College of Wisconsin - Central Wisconsin Campus, UNITED STATES

## Abstract

A solid understanding of basic sciences is a prerequisite for successful completion of medical education. Therefore, it is essential to improve the quality of teaching and to ensure the applicability of basic sciences. Based on practical experiences and previous research, we developed an innovative step-by-step concept, called ENHANCE, for the implementation or revision of teaching units, especially for basic sciences. We used comparative self-assessment gains, a questionnaire to assess teaching quality as well as end-of-semester evaluations (students’ satisfaction and open-ended questions) to evaluate the ENHANCE concept. It was found that ENHANCE-based teaching units were related to increased students’ satisfaction, high attendance rates and that restructuring the course curriculum yielded in a positive assessment of teaching effectiveness. The revised courses were rated as the very best of all classes in several semesters. Qualitative data showed that students particularly appreciated the level of comprehension and how helpful the courses were for the understanding and preparation of the regular curriculum.

## Introduction

A sound knowledge and understanding of basic sciences are a prerequisite for a successful completion of medical education. Fundamental knowledge in basic sciences serves as a major determinant of diagnostic success, because numerous modern devices and methods used for diagnostics and treatments are based on these [1, 2]. Nevertheless, there is a significant variance in students’ basic science knowledge at the beginning of medical education [3]. One reason for this variance in knowledge is the large heterogeneity in secondary education and the fact that students are not obliged to take basic science courses like chemistry or physics during their last years in high school in Germany. Insufficient knowledge in basic sciences can lead to a lack of understanding of fundamental biochemical, physiological, and pathophysiological principles and possibly to a dropout of students [4]. Due to this challenging task, medical schools developed different methods to prepare their students for the medical curriculum. For instance, pre-medical courses are offered in many countries [5, 6].

### Integrated supportive science courses in the reformed medical curriculum iMED

An integrated supportive science (ISS) course program was developed at the University Medical Center Hamburg-Eppendorf to teach sciences (chemistry, biology, physics, and mathematics). This course concept is *longitudinally* included in the reformed medical curriculum iMED, which is characterized by a connection of pre-clinical and clinical learning contents and a continuously increasing proportion of clinical topics. The iMED curriculum consists of a modular compulsory core curriculum that is comprised of 19 modules. Seven module groups cover three stages of a learning spiral. Each of the first nine semesters consist of two six-week compulsory modules and one two-week elective module (for further information, see [7]).

The ISS courses are matched to the needs of students and the core curriculum. They are taught *alongside* the iMED curriculum and aim to teach basic science concepts and fundamental knowledge. At the beginning of the curriculum, the ISS courses focus on basic knowledge that is essential to prepare students for topics of the upcoming semesters. For example, the musculoskeletal system is one of the main topics. Therefore, basic knowledge of electricity and mechanics are taught in the physics ISS courses to prepare for physiology classes. The ISS courses are integrated into the core curriculum up until the sixth or seventh semester. This approach was chosen because many higher level students wished that they would have paid more attention during the first two years of ISS courses, as they finally appreciated the importance of these courses in higher semesters [8].

In a previous study, we showed that the temporal and content-based integration of the ISS courses into the medical curriculum promotes the understanding of basic science concepts and enhances the motivation to acquire basic science knowledge [9]. The students in our study reported better maintenance of basic science knowledge after ISS course participation [9]. Based on qualitative and quantitative data from course evaluations, we continuously adjusted and optimized our course program.

### Aims of the study

Only little research has been conducted regarding ISS course programs during medical education. At our institution, results from our previous study showed that the satisfaction of students regarding the ISS program could be improved by taking the perspective of students seriously and by focusing on topics that are relevant from a medical point of view. Strong instructional program coherence stems from the use of an integrated framework that “combines specific expectations for student learning with specific strategies and materials to guide teaching and assessment” [10]. In the present study, the primary aim was to explore the reasons for students’ dissatisfaction, identify incoherence and find ways to further improve the ISS course program. Further aims were to capture the students’ self-assessments of the intended learning outcomes (ILOs), and to assess teaching quality in detail in selected ISS courses. In order to disseminate the instructional design concept of our course program, we developed a seven-step approach, called ENHANCE, which is an innovative, coherent, and integrated concept for the development and revision of teaching units, especially for basic sciences in medical education. In this paper, we describe the concept and report mixed-methods evaluation results that were collected before, during, and after the implementation of ENHANCE.

## Materials and methods

### The ENHANCE concept

**Fig 1:**
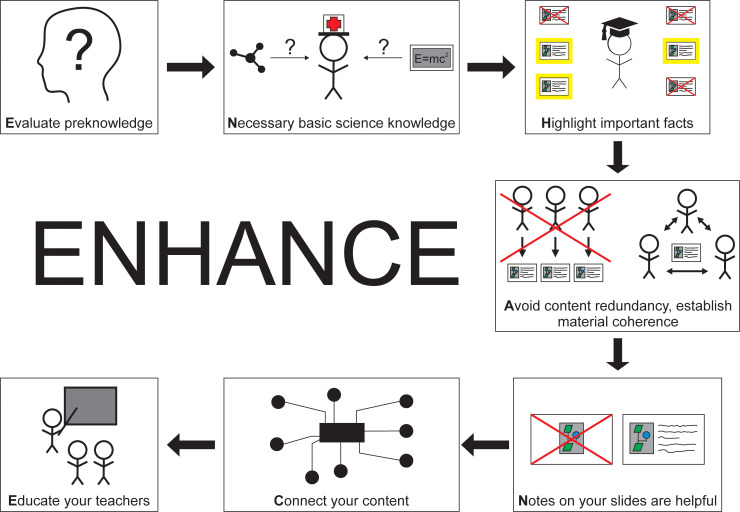
This figure shows the seven steps of the ENHANCE concept.

The ENHANCE concept is a seven-step approach that may be a useful tool for the development and revision of teaching units and is described in detail in the following seven paragraphs (Fig 1).

1. **Evaluate preknowledge:** *What kind of basic science knowledge do medical students bring along at the beginning of medical education*?
  Because of the heterogeneity of students’ preknowledge, we recommend assessing what level of knowledge can be expected from beginning students by screening school curricula and interviewing the students. Gaps in knowledge can be identified by comparing these results and a list of topics that build the necessary foundation of basic sciences in medical education.
2. **Necessary basic science knowledge:** *What kind of basic science knowledge is necessary for a solid understanding of clinical subject areas*?
  Often, teachers of subject areas like physics, chemistry, or biology do not have a medical background [11]. As a result, most of the course materials are lacking examples with medical relevance but rather focus on higher order concepts and details. In our ISS course program, an experienced medical student (first author, KK) was involved in strengthening the connection between the needs and knowledge of the students and the existing ISS courses. All necessary contents were linked to subject areas like physiology, biochemistry, and other clinical subjects in this process.
3. **Highlight important facts:** *Let it go–physicists love physics; medical doctors love medicine*: *find the happy medium*.
  Cognitive load theory suggests that teachers should not focus on details because students learn less when lectures contain too much information [12]. This might be difficult, as every teacher has enthusiasm for their subject. During the development of the ISS courses, subject-specific details were reduced, and the needs of the medical students regarding physics, chemistry, biology, and mathematics content were focused on. It is key to present an overview to develop a knowledge base, and to focus on details afterwards.
4. **Avoid content redundancy, establish material coherence:** *Less is more–use the same figures and charts*.
  The teachers from different subject areas are challenged to cooperate when designing a coherent curriculum to avoid content redundancy (on the advantages of instructional program coherence, see [10]). It is recommended to apply standards for the reuse of the same figures, tables, and charts across different subject areas to establish material coherence. For example, the same figure of the electric dipole of the heart can be used in physics, physiology, and cardiology, which will result in recognition and therefore better retention. This also demonstrates how basic concepts of sciences are used across different subject areas and highlights the importance of these topics for the upcoming more advanced clinical subjects [13].
5. **Notes on your slides are helpful:** *Use the space on your slides for explanations*.
  An integrated curriculum makes it difficult for students to process many different subjects at the same time [9]. Students use slides to study independently and are thankful if they contain explanations to understand the figures, charts, and tables even though it has been shown that it might be not useful to put full sentences and too much text on lecture slides [14]. That is why lecture slides should not contain too much text, but some explaining words and key messages. To avoid distractions during the lecture, the text can be hidden, and a text-version can be uploaded afterwards, preferably with recommendations for further readings.
6. **Connect your content:** *Link your content to the content of other subjects–even if these are going to be taught later*.
  It is recommended to use footnotes on slides to explicitly point out for which topic or level of the curriculum the presented content is needed. Students will be more willing to invest time to learn a topic after understanding the topic’s relevance and will be more satisfied when learning complex contents [15]. For example, the physical basics of sonography can be taught before students see a demonstration of the cardiac cycle by means of echocardiography. Additionally, the enthusiasm of teachers to design an integrated curriculum as well as cooperation across departments may lead students to better understand how different subject areas are connected [16].
7. **Educate your teachers:** *Implementation of mandatory teacher training*.
  The use of slides and materials that were not developed by the teachers themselves might be challenging for them. Therefore, it is advisable to train staff members continuously [17]. Additionally, all teachers should meet and discuss each course before the start of the semester. This reduces ambiguities, allows modifications, and reduces misunderstandings.
  Homogeneity of lecture material is highly recommended because each teacher might present slightly different topics in a different manner.

### Procedure

Formative and summative evaluation methods were used to assess the ISS courses since the beginning of their implementation in 2012. A team of researchers and teachers focused on the improvement of the physics ISS courses. The revision of the following physics ISS courses was started in 2015: mechanics (semester 1), electricity 1 (semester 1), electricity 2 (semester 1), pressure & fluid mechanics (semester 1), radiation 1 (semester 6/7), and radiation 2 (semester 6/7). Over a period of two years, the step-by-step approach ENHANCE was designed and continuously revised to improve these physics ISS courses. Also, four new physics ISS courses were developed during this phase: basics of X-radiation (semester 1), exercise and question time 1 & 2 (semester 1) and basics of sonography (semester 1).

A mixed-methods approach was used to assess the success of this revision process using the following tools:

- Study 1: Summative semester evaluation (judgement of satisfaction, open-ended questions)
- Study 2: Comparative self-assessment (CSA) of ILOs [18, 19] and the Student Evaluation of Teaching in MEDical Lectures (SETMED-L) questionnaire [20].

#### Participants study 1

The standard summative evaluation of the Medical Faculty was used to quantitatively and qualitatively assess students’ satisfaction. Participation in this type of evaluation is voluntary and anonymous but highly encouraged. In every module, response rates are high (> 90%). Each year, approximately 350–400 students are admitted to the program. In their first year, the whole cohort is invited to participate in the ISS courses. In the advanced modules, cohorts are split into four groups to ensure small-group learning resulting in smaller samples in the summative evaluation. The number of participants (n) in the individual course evaluations is presented in S1 Table. Judgements of students’ satisfaction regarding the physics ISS courses were collected in the period between winter semester (WS) 2013/14 and WS 2019/20.

#### Participants study 2

CSA and teaching quality were assessed in an online-survey using the EvaSys software (Electric Paper Evaluations systeme, licensed by the University of Hamburg). At the end of each course in WS 2018/19, students were asked to complete the survey using their smartphones. Respondents were assured of anonymity of their data. On average, response rates were around 40%. Participants were 371 (70% female; 259 of 371) medical students who voluntarily participated in one of the following physics ISS courses: radiation 1 (n = 34), radiation 2 (n = 22), basics of sonography (n = 83), basics of X-radiation (n = 158), and pressure & fluid mechanics (n = 74). All courses investigated in Study 2 were taught by the same lecturer according to the ENHANCE concept, some of them as a lecture for the whole semester, others in smaller groups.

### Materials

#### Judgement of satisfaction

Judgements of satisfaction were assessed of all participants in the summative online evaluation at the end of each module. For each course, students were asked to rate the following statement on a six-point Likert scale ranging from (1 = totally disagree to 6 = totally agree): “I am overall satisfied with the teaching in [discipline, type of course].”. Additionally, an open-ended question was integrated in this questionnaire to better understand quantitative ratings.

#### Outcome-based evaluation: Teaching effectiveness

The validated CSA tool was used to investigate teaching effectiveness. Students rated their initial knowledge in terms of specific ILOs retrospectively as well as their current level of knowledge after the course. The development and design of the method as well as the validation of the CSA tool is extensively described elsewhere [18, 19, 21, 22]. The CSA gain served as an indicator of the relative increase in knowledge regarding pre-defined ILOs. ILOs were formulated during the revision of the ISS courses according to the ENHANCE concept. At the end of each session, students were asked to comparatively rate four to eight statements or rather individual ILOs on a six-point Likert scale (extremes only labeled; 1 = fully agree, 6 = completely disagree). They were asked to rate their knowledge at the end of the session (post-test rating) and before the session (then-test rating). For example, students were asked to rate the following ILOs: “I can safely reproduce the functioning of an ultrasound transducer in its essentials.” or “I can easily explain relevant sources of error/influencing factors in the formation of a radiographic image.”. The comparison and conversion of initial and final self-assessments results in gain scores ranging from -100% to +100%. The validation study of the CSA tool showed that gains were highly correlated with results of an objective examination [21].

#### Teaching quality

Teaching quality was assessed using a 14-item questionnaire that has been developed to specifically evaluate lectures in medical education [20]. The questionnaire consists of one major factor (“Core teaching skills”, ten items, e.g., “Session is well structured”) and two minor factors (“Student activation skills”, two items, e.g., “Teacher asks questions to check student learning outcome”; “Student workload”, two items, e.g., “Teaching pitched to the student level”). A five-point Likert scale was used for responses: “strongly disagree” (1), “disagree” (2), “neither agree nor disagree” (3), “agree” (4), and “strongly agree” (5). Cronbach’s alpha coefficients as a measure of internal consistency of the three subscales were sufficient for two of the three subscales: α = 0.723 (“Student workload”) and α = 0.816 (“Core teaching skills”). Due to insufficient internal consistency of the “Student activation” factor (α = 0.583), both items in this factor were assessed separately.

### Data analyses

Judgements of satisfaction were descriptively analyzed in each physics ISS course from the beginning of WS 2013/14. The Kolmogorov-Smirnov test was used to see if the distribution of data significantly differed from a normal distribution. Due to the large number of different classes, investigated ISS courses were grouped in three different categories: Condition 1 = courses taught without a consistent concept (non-ENHANCE); condition 2 = courses partly taught according to the ENHANCE concept (part-ENHANCE); condition 3 = courses taught according to the ENHANCE concept (total-ENHANCE). Differences in students’ satisfaction were assessed using the Kruskal-Wallis test since skewed distributions were to be expected. Results were followed up using Mann-Whitney tests. A Bonferroni correction was applied during this procedure resulting in a significance level of 0.0167. Additionally, a ranking of all judgements of satisfaction for all courses that were taught in each semester was built to compare the rating of the ISS courses to all other courses (Study 1). Further, all comments by the students in the summative evaluation from WS 2015/16 to summer semester (SS) 2019 were analyzed using a mix of deductive and inductive category formation in MAXQDA 2018 (VERBI GmbH). Initially, a coding frame of three top-level codes was built: positive feedback, negative criticism and suggestions for improvement. Specific themes associated with one of these codes were defined in an inductive process based on the comments themselves. All documents were completely worked by one coder through to build categories representing all themes. In a next step, the category system was partly revised. In this process, all codes in all categories were screened. Discrepancies were solved by reattributing codes to the more appropriate category. The aim of this step was to clearly differentiate and consolidate ambiguous categories.

In Study 2, differences in the teaching quality of the ISS courses were assessed using the Kruskal-Wallis test since skewed distributions, and ceiling effects were to be expected [20]. In the literature, different thresholds for ceiling effects are reported. Accordingly, ceiling effects are present if > 15% [23] or > 20% [24] of participants choose the maximum possible score of an item. An alpha level of 0.05 was used for this statistical test.

For the CSA data, the learning-outcome specific gain was computed using the following formula:

$$CSA\;gain\;(\%)=\frac{\mu_{pre}-\mu_{post}}{\mu_{pre}-1}∙100$$

where *μ_pre_* is the mean initial self-assessment and *μ_post_* the mean self-assessment after the course.

### Ethical approval

The study was realized in accordance with the Declaration of Helsinki. When entering medical education at the University Medical Center Hamburg-Eppendorf, students are required to officially consent to the participation and use of the web-based evaluation. The consent is voluntary, and over 99% of students give it. The anonymity of participants is guaranteed.

The research protocol was reviewed and approved by the Dean of the Medical Faculty since there was no institutional ethics committee and the State Medical Association only reviews biomedical or epidemiological research.

## Results

### Study 1

From March 2014 to March 2020, a total of 31 semester evaluations were collected. Six preexisting ISS courses (mechanics; electricity 1 & 2; pressure & fluid mechanics; radiation 1 & 2) have been revised and four ISS courses (basics of X-radiation; exercise and question time 1 & 2; basics of sonography) have been additionally implemented in the curriculum. Students’ satisfaction was assessed in every course (see S1 Table). Descriptive statistics of the three different conditions showed a positive trend in students’ satisfaction (non-ENHANCE: size of the sample (n) = 1476; mean (M) = 4.37; standard deviation (SD) = 1.32; part-ENHANCE: n = 1020; M = 4.67; SD = 1.22; total-ENHANCE: n = 2116; M = 5.57; SD = 0.83). In order to statistically compare the three different conditions, a non-parametric Kruskal-Wallis test was performed, because results of the Kolmogorov-Smirnov test showed a significant departure form normality. Students’ satisfaction was significantly affected by the concept, *H*(2) = 1146.316, p < 0.001. Mann-Whitney tests were used to follow up this finding. The three groups were compared to each other in three tests. Results of all three comparisons were highly significant. The most positive rating was found for the total-ENHANCE condition.

Results of the rankings of all courses showed that students were repeatedly most satisfied with some of our physics ISS courses: “radiation 1 & 2” (SS 2017, WS 2017/18 and SS 2018) as well as “basics of sonography” (WS 2018/19) were at the top of the ranking of around 450 teaching units each semester. In WS 2018/19 the course “radiation 1 & 2” came in second in the ranking.

Table 1 presents the total number of students who commented on the physics ISS courses, the number of themes that were identified in these comments, and the absolute and relative number of positive feedback and negative criticism. The third top-level code is not integrated in this table, because very few students gave concrete suggestions for improvement (codings n = 8).

**Table 1:** Number of comments, themes, positive feedback, and negative criticism for all physics integrated supportive science courses.

	Number of students who commented	Number of themes	Positive Feedback (absolute; relative)	Negative Criticism (absolute; relative)
Courses taught without a consistent concept (non-ENHANCE)				
WS 2015/16 (Sem. 1)	15	14	3; 21%	8; 57%
SS 2016 (Sem. 7)	11	15	11; 73%	4; 27%
WS 2016/17 (Sem. 1)	24	25	10; 40%	15; 60%
Total	50	54	24; 44%	27; 50%
Courses partly taught according to the ENHANCE concept (part-ENHANCE)				
WS 2015/16 (Sem. 6)	10	12	10; 83%	2; 17%
WS 2016/17 (Sem. 6)	12	15	14; 93%	1; 7%
WS 2017/18 (Sem. 1)	47	57	34; 60%	23; 40%
Total	69	84	58; 69%	26; 31%
Courses taught according to the ENHANCE concept (total-ENHANCE)				
SS 2017 (Sem. 7)	21	27	27; 100%	0; 0%
WS 2017/18 (Sem. 1)	51	69	51; 74%	17; 25%
WS 2017/18 (Sem. 6)	17	23	20; 87%	3; 13%
SS 2018 (Sem. 7)	27	41	37; 90%	0; 0%
WS 2018/19 (Sem. 6)	11	20	20; 100%	0; 0%
WS 2018/19 (Sem. 1)	79	144	129; 90%	15; 10%
WS 2018/19 (Sem. 1)	32	54	51; 94%	3; 6%
SS 2019 (Sem. 7)	10	15	14; 93%	1; 7%
Total	248	393	349; 89%	39; 10%

Sem., semester; SS, summer semester; WS, winter semester. Response percentages may not add up to 100% due to very few codings in the category “Suggestions for improvement”.

During the implementation phase of the ISS courses, the ENHANCE concept was not thoroughly realized in every ISS course. The fact that negative criticism was more frequent in the first two and a half years corroborates the advantages of this concept. Specifically, negative criticism in the first two and a half years included predominantly the following themes: general criticism of the lecturer (codings n = 15), amount of content too big (codings n = 11), criticism of lecturer behavior to overcome difficulties (codings n = 9). For example, two students wrote:

“The lecturer wasn’t able to transfer his knowledge effectively to me. When questions came up, he answered scarcely and ambiguously.” (WS 2015/16)

“The amount of information that was supposed to be taught was unfortunately way too much for the limited amount of time.” (WS 2017/18)

When the ENHANCE concept was implemented in every physics ISS course, the positive feedback by far outweighed the negative criticism. The most frequent theme was general praise of the lecturer (codings n = 113), for example “The lecturer did a great job.” (SS 2017). Many students also praised the level of comprehension achieved in the courses (codings n = 58), for example “The course managed to explain difficult matters in a comprehensible way.” (WS 2018/19). Another important theme was the emphasis on how helpful the ISS courses were for the understanding and preparation of the regular curriculum (codings n = 47). Many students gave general words of appreciation (codings n = 38, e.g., “The ISS courses are great!” SS 2018). Students also specifically commented on the ENHANCE concept (codings n = 34). In these comments, students either gave some general feedback (e.g., “This concept made it fun to learn physics, and this is quite a surprise.” WS 2018/19), or explicitly pointed out the advantages of the concept (e.g., “This course was great. The teacher did not go into too much detail, but rather covered the essentials. The teacher was very motivated and able to convey the material vividly.” SS 2018).

### Study 2

Teaching quality was assessed by means of the SETMED-L questionnaire in all physics ISS courses in WS 2018/19. Descriptive statistics of “Core Teaching Skills”, “Student Workload”, as well as both items regarding “Student Activation Skills” of all five physics ISS courses are presented in Table 2. Results of the Kolmogorov-Smirnov test showed a significant departure form normality in all scores. Accordingly, ceiling effects were detected in all items: 51–95% of participants chose the highest possible score and showed a strong agreement.

**Table 2:** Descriptive statistics of the SETMED-L factors/items.

	Basics of sonography		Pressure & fluid mechanics		Radiation 1		Radiation 2		Basics of X-radiation	
	n	Mdn	n	Mdn	n	Mdn	n	Mdn	n	Mdn
Teaching Skills	83	5	74	4.9	33	4.8	22	4.8	157	4.9
Student Workload	83	4.5	74	4.5	33	4	22	4.5	157	5
Teacher asks questions	83	4	74	5	33	5	22	5	157	4
Teaching/ Participation	83	4	74	4	33	5	22	5	157	5

n, sample; Mdn, median.

Significant differences between the physics ISS courses were detected regarding the “Core Teaching Skills” (*H*(4) = 14.96, *p* = 0.005), “Student Workload” (*H*(4) = 20.42, *p* < 0.001) and the item “Teacher asks questions to check student learning outcome” (*H*(4) = 24.93, *p* < 0.001). Differences between the physics ISS courses regarding the item “Adequate balance between didactic teaching and student participation” were not significant (*H*(4) = 7.19, *p* = 0.126).

### Comparative self-assessment

Teaching effectiveness was assessed using the CSA tool. Large CSA gains were calculated for all ILOs. The most homogenous gain in all ILOs was found in the physics ISS course “basics of X-radiation”. The largest gain variation of the different ILOs was detected in the physics ISS course “pressure & fluid mechanics”. M, SD, minimum and maximum CSA gains as well as then- and post-test M and SD of the ILOs are reported in Table 3.

**Table 3:** Comparative self-assessment gains and intended learning outcomes ratings at then- and post-test of five different physics integrated supportive science courses.

	CSA gain (%)			ILO ratings	
	M (SD)	Min	Max	Then-test: M (SD)	Post-test: M (SD)
Basics of X-radiation	71% (1%)	70%	73%	4.63 (0.29)	2.06 (0.08)
Radiation 1	67% (5%)	57%	71%	4.73 (0.28)	2.24 (0.19)
Radiation 2	69% (6%)	62%	78%	5.15 (0.18)	2.31 (0.31)
Pressure & fluid mechanics	68% (7%)	60%	82%	4.39 (0.59)	2.12 (0.40)
Basics of sonography	71% (4%)	67%	78%	5.08 (0.51)	2.20 (0.22)

CSA, comparative self-assessment; ILO, intended learning outcome; M, mean; Max, maximum; Min, minimum; SD, standard deviation.

## Discussion

In this paper, we describe the development, evaluation and implementation of the instructional design concept ENHANCE. To test the effectiveness of the concept, we collected qualitative and quantitative judgements of satisfaction as well as ratings of teaching effectiveness (CSA) and quality. Data were collected over the course of six years in which ISS courses were revised or newly developed to meet student needs. In sum, results show that the use of the ENHANCE concept significantly increased students’ satisfaction (Study 1) and that restructuring the course curriculum yielded in a positive assessment of teaching quality and effectiveness (Study 2). Below, results are discussed in detail, especially regarding the physics ISS courses radiation 1 & 2.

The physics ISS courses radiation 1 & 2 were first taught in the SS 2015. Initially, students’ satisfaction was good (M = 4.81, SD = 0.73), but attendance rates were below 10% in both courses. Due to this, a major course revision following the ENHANCE concept was initiated. All students in the relevant semesters were informed at the beginning of the study period about the revision of the course program. In line with the results of Newmann and colleagues [10] the identification of incoherence and the implementation of a common instructional framework resulted in different benefits: First, attendance rates increased up to 70% which corroborates findings of Billings-Gagliardi and Mazor [25] who found that medical students’ attendance decisions are mostly deliberate based on their estimation of how beneficial a lecture might be for their learning. Further, the global judgements of satisfaction rose up to ratings of M = 5.92 on a six-point scale. Qualitative data showed that students appreciated the fact that the ISS courses were helpful to prepare for the regular classes and that the material was coherent and comprehendible.

Study 2 was carried out to assess five of the physics ISS courses in detail. A high agreement was found regarding the teaching effectiveness (“Teaching skills”, “Student workload”, “Teacher asks questions”, and “Teaching/participation”). The agreement regarding the sub-scales “Teacher asks questions” and “Teaching/participation” was significantly higher in smaller courses (radiation 1 & 2: maximum group size around 50 students) than in courses with more than 100 students (Table 2). In the validation study of the instrument, the authors found very high ratings regarding the items on student activation in large group lectures [18]. From a didactical point of view, the difference found in the present study seems reasonable, because teachers and students interact more directly in small groups [26]. The agreement regarding the student workload was higher in courses with a high share of medical topics and crosslinks to other subjects. This could be attributed to the fact that connected learning experiences are beneficial to student learning and engagement in coherent instructions [8]. Also, clinical correlations are explicitly used in basic science teaching to foster student interest and show relevance [27]. A few differences in the CSA gains regarding the ILOs were identified. Two different observations might be relevant at this point: First, some of the ILOs with lower CSA gains were not prioritized in the particular course due to several reasons (either the topic was taught before or the topic was going to be taught in the near future in more detail). Second, students were already familiar with some of the topics which resulted in high self-assessments in the then-test rating. Using the CSA tool, we identified some ILOs that were not represented adequately in the curriculum. As a consequence, the teaching units were restructured. Altogether, the CSA tool is useful to identify contents that are not adequately covered. This tool cannot be used to assess actual learning of students. Following our approach of designing a coherent instruction, it can be used as a formative feedback tool to improve courses.

Besides these quantitative analyses of the ISS course program, the attendance rates strongly demonstrate that the students are highly satisfied with the voluntary ISS courses. As mentioned before, some of our ISS courses repeatedly achieved the highest students’ satisfaction ratings of the whole semester. This fact should be highlighted, as students in the sixth or seventh semester usually are more fascinated by clinical teaching rather than by a voluntary physics ISS course. Qualitative data showed that 20 students explicitly wrote that they had fun in the course.

Educators have to face the challenge that students suffer from a significant loss of knowledge after completing a course. It was found that medical students retained only 47% of their previously acquired knowledge in basic science courses [28]. Future studies have to assess whether students being taught according to the ENHANCE concept will be able to retain more of the acquired basic science knowledge.

## Limitations

This study has potential limitations. Due to a complex rotation schedule of the iMED curriculum and the anonymity of data, we were not able to merge data in Study 1. Accordingly, the same cohorts evaluated all of the different ISS courses that they visited, but we were not able to track possible changes in their satisfaction. In Study 2, the same teacher (KK) taught all ISS courses that were analyzed in detail using the CSA tool and SETMED-L questionnaire due to two reasons. First, possible differences in the CSA gain that could be attributed to different teachers should be avoided. Second, only one teacher with a medical background was available to teach in the program. Results of Study 1 already showed that physics ISS courses taught based on the ENHANCE concept were more positively rated than courses without a consistent concept. Nevertheless, a comparison to other ISS courses that are not based on the ENHANCE concept would add to the validation of the concept.

Furthermore, strong ceiling effects were detected in the present study. The problem of ceiling effects was also detected during the design and validation of the SETMED-L questionnaire [20], and the question came up whether ceiling effects could be eliminated if a sufficient range of the scale would be established to produce variability [29]. The fact that only one teacher was assessed in the present study made it difficult to discriminate whether the ceiling effect occurred because of the non-sufficient measurement design or because the teaching quality of this single teacher actually was excellent.

Besides the fact that the CSA tool is a self-report tool, it has some practical limitations. Using the tool correctly, only one specific ILO is assessed with each item. Some of the teaching units of the present ISS courses contain up to 18 specific ILOs. In order to reduce the time and effort for students and to construct an effective questionnaire, a few related ILOs were merged and covered with one item. This procedure was in some cases also used by the originators of the tool [18].

Objective measurements of students’ knowledge gain could not be conducted due to the following reasons: First, subject-specific questions do not exist in the assessment at our institution. All questions refer to different subjects, for example, physiology, physics, and cardiology. Therefore, it is not possible to attribute the correct answer in the final examination in each module to a specific ISS course. Second, all data regarding the teaching effectiveness and the CSA gain were anonymous and could not be linked to the final results of the exams.

The format of Likert scales differed between the studies. Although this requires the reader to carefully consider the differences when interpreting the results, it was decided to keep the validated format of each scale.

## Conclusion

A sound understanding of basic sciences is key for the successful completion of medical education. It has been shown that a fundamental knowledge of basic sciences is a major determinant of diagnostic success [1, 2]. Therefore, it is indispensable to improve the quality of teaching and to meet the needs of medical students in terms of usefulness, especially in basic sciences. ENHANCE is an innovative, coherent, and integrated approach for the development and revision of teaching units for medical students, especially for basic sciences. In our study, we showed that ENHANCE-based teaching units lead to an increased students’ satisfaction and that restructuring the course curriculum yielded in a positive assessment of teaching quality and effectiveness. We believe that this concept can easily be applied in a different context to facilitate interaction with regard to content alignment among teaching staff.

## Supporting information

S1 Table Satisfaction in all physics integrated supportive science courses since 2013. n, sample; M, mean; SD, standard deviation; SS, summer semester; WS, winter semester.
(PDF)

## References

[1] Rotomskis R, Karenauskaite V, Balzekiene A. Biomedical physics in continuing medical education: an analysis of learning needs. Medicina (Kaunas). 2009;45(11):918–28. 20051725

[2] Woods NN, Neville AJ, Levinson AJ, Howey EH, Oczkowski WJ, Norman GR. The value of basic science in clinical diagnosis. Acad Med. 2006;81(10 Suppl):S124–7. 17001122 10.1097/00001888-200610001-00031

[3] von Dülmen M KC, Ludwig R, Schulze J. Naturwissenschaftliche Vorkenntnisse deutscher Studienanfänger in der Humanmedizin. GMS Z Med Ausbild. 2006;23.

[4] Arulampalam W, Naylor R, Smith J. Factors affecting the probability of first year medical student dropout in the UK: a logistic analysis for the intake cohorts of 1980–92. Med Educ. 2004;38(5):492–503. 10.1046/j.1365-2929.2004.01815.x 15107083

[5] Schneid SD, Apperson A, Laiken N, Mandel J, Kelly CJ, Brandl K. A summer prematriculation program to help students succeed in medical school. Adv Health Sci Educ Theory Pract. 2018;23(3):499–511. 10.1007/s10459-017-9808-8 29340892

[6] Wilson WA, Henry MK, Ewing G, Rehmann J, Canby CA, Gray JT, et al A prematriculation intervention to improve the adjustment of students to medical school. Teach Learn Med. 2011;23(3):256–62. 10.1080/10401334.2011.586923 21745061

[7] Rheingans A, Soulos A, Mohr S, Meyer J, Guse AH. The Hamburg integrated medical degree program iMED. GMS J Med Educ. 2019;36(5):Doc52 10.3205/zma001260 31815162 PMC6883244

[8] Spencer AL, Brosenitsch T, Levine AS, Kanter SL. Back to the basic sciences: an innovative approach to teaching senior medical students how best to integrate basic science and clinical medicine. Acad Med. 2008;83(7):662–9. 10.1097/ACM.0b013e318178356b 18580085

[9] Eisenbarth S, Tilling T, Lueerss E, Meyer J, Sehner S, Guse AH, et al Exploring the value and role of integrated supportive science courses in the reformed medical curriculum iMED: a mixed methods study. BMC Med Educ. 2016;16:132 10.1186/s12909-016-0646-9 27129494 PMC4851779

[10] Newmann FM, Smith B, Allensworth E, Bryk AS. Instructional Program Coherence: What It Is and Why It Should Guide School Improvement Policy. Educ Eval Policy Anal. 2001;23(4):297–321.

[11] Weston WW. Do we pay enough attention to science in medical education? Can Med Educ J. 2018;9(3):e109–e14. 30140355 PMC6104321

[12] Young JQ, Van Merrienboer J, Durning S, Ten Cate O. Cognitive Load Theory: implications for medical education: AMEE Guide No. 86. Med Teach. 2014;36(5):371–84. 10.3109/0142159X.2014.889290 24593808

[13] Vuletic L, Spalj S, Peros K. Visual presentation of a medical physiology seminar modifies dental students' perception of its clinical significance. Eur J Dent Educ. 2016;20(1):14–9. 10.1111/eje.12132 25490947

[14] Prasad S, Roy B, Smith M. The art and science of presentation: electronic presentations. J Postgrad Med. 2000;46(3):193–8. 11298471

[15] Brauer DG, Ferguson KJ. The integrated curriculum in medical education: AMEE Guide No. 96. Med Teach. 2015;37(4):312–22. 10.3109/0142159X.2014.970998 25319403

[16] Dahle LO, Brynhildsen J, Behrbohm Fallsberg M, Rundquist I, Hammar M. Pros and cons of vertical integration between clinical medicine and basic science within a problem-based undergraduate medical curriculum: examples and experiences from Linkoping, Sweden. Med Teach. 2002;24(3):280–5. 10.1080/01421590220134097 12098414

[17] Malik AS, Malik RH. Twelve tips for developing an integrated curriculum. Med Teach. 2011;33(2):99–104. 10.3109/0142159X.2010.507711 20874013

[18] Raupach T, Munscher C, Beissbarth T, Burckhardt G, Pukrop T. Towards outcome-based programme evaluation: using student comparative self-assessments to determine teaching effectiveness. Med Teach. 2011;33(8):e446–53. 10.3109/0142159X.2011.586751 21774642

[19] Raupach T, Schiekirka S, Munscher C, Beissbarth T, Himmel W, Burckhardt G, et al Piloting an outcome-based programme evaluation tool in undergraduate medical education. GMS Z Med Ausbild. 2012;29(3):Doc44 10.3205/zma000814 22737199 PMC3374140

[20] Muller T, Montano D, Poinstingl H, Dreiling K, Schiekirka-Schwake S, Anders S, et al Evaluation of large-group lectures in medicine—development of the SETMED-L (Student Evaluation of Teaching in MEDical Lectures) questionnaire. BMC Med Educ. 2017;17(1):137 10.1186/s12909-017-0970-8 28821257 PMC5563045

[21] Schiekirka S, Reinhardt D, Beißbarth T, Anders S, Pukrop T, Raupach T. Estimating learning outcomes from pre- and posttest student self-assessments: a longitudinal study. Acad Med. 2013;88(3):369–75. 10.1097/ACM.0b013e318280a6f6 23348083

[22] Schiekirka S, Anders S, Raupach T. Assessment of two different types of bias affecting the results of outcome-based evaluation in undergraduate medical education. BMC Med Educ. 2014;14:149 10.1186/1472-6920-14-149 25043503 PMC4112834

[23] McHorney CA, Tarlov AR. Individual-patient monitoring in clinical practice: are available health status surveys adequate? Qual Life Res. 1995;4(4):293–307. 10.1007/BF01593882 7550178

[24] Wang L, Zhang Z, McArdle JJ, Salthouse TA. Investigating Ceiling Effects in Longitudinal Data Analysis. Multivariate Behav Res. 2009;43(3):476–96. 10.1080/00273170802285941 19924269 PMC2778494

[25] Billings-Gagliardi S, Mazor KM. Student decisions about lecture attendance: do electronic course materials matter? Acad Med. 2007;82(10 Suppl):S73–6. 17895696 10.1097/ACM.0b013e31813e651e

[26] Edmunds S, Brown G. Effective small group learning: AMEE Guide No. 48. Med Teach. 2010;32(9):715–26. 10.3109/0142159X.2010.505454 20795801

[27] Klement BJ, Paulsen DF, Wineski LE. Clinical Correlations as a Tool in Basic Science Medical Education. J Med Educ Curric Dev. 2016;3:JMECD.S18919. 10.4137/JMECD.S18919 PMC5758745 29349328

[28] D'Eon MF. Knowledge loss of medical students on first year basic science courses at the University of Saskatchewan. BMC Med Educ. 2006;6:5 10.1186/1472-6920-6-5 16412241 PMC1397826

[29] Keeley JW, English T, Irons J, Henslee AM. Investigating Halo and Ceiling Effects in Student Evaluations of Instruction. Educ and Psychol Meas. 2013;73(3):440–57.

