# Supplementary material for: Presentation and evaluation of the teaching concept “ENHANCE” for basic sciences in medical education
Source: PLoS One. 2020 Sep 29;15(9):e0239928. doi: 10.1371/journal.pone.0239928 (PMC7523967; doi:10.1371/journal.pone.0239928)
Supplement: S1 Table — n, sample; M, mean; SD, standard deviation; SS, summer semester; WS, winter semester. (PDF) [file pone.0239928.s001.pdf]

**Supplementary Table S1. Satisfaction in all physics integrated supportive science courses since 2013.**

| | Exercise/question time 1 & 2 | | | Mechanics, Electricity 1 & 2 | | | Basics of X-radiation | | | Pressure & fluid mechanics | | | Basics of sonography | | | Radiation 1 & 2 | | |
|---|---|---|---|---|---|---|---|---|---|---|---|---|---|---|---|---|---|---|
| | n | M | SD | n | M | SD | n | M | SD | n | M | SD | n | M | SD | n | M | SD |
| Courses taught without a consistent concept (non-ENHANCE) | | | | | | | | | | | | | | | | | | |
| WS 2013/14 | | | | 286 | 3.61 | 1.44 | | | | 175 | 4.41 | 1.15 | | | | | | |
| WS 2014/15 | | | | 274 | 4.34 | 1.25 | | | | 185 | 4.63 | 1.25 | | | | | | |
| SS 2015 | | | | | | | | | | | | | | | | 21 | 4.81 | 0.75 |
| WS 2015/16 | | | | | | | | | | 183 | 4.47 | 1.07 | | | | | | |
| SS 2016 | | | | | | | | | | | | | | | | 38 | 4.97 | 1.20 |
| WS 2016/17 | | | | | | | | | | 142 | 4.75 | 1.31 | | | | | | |
| WS 2017/18 | | | | | | | | | | 172 | 4.77 | 1.22 | | | | | | |
| Courses partly taught according to the ENHANCE concept (part-ENHANCE) | | | | | | | | | | | | | | | | | | |
| WS 2015/16 | | | | 319 | 4.25 | 1.24 | | | | | | | | | | 46 | 5.43 | 1.03 |
| WS 2016/17 | | | | 283 | 4.69 | 1.18 | | | | | | | | | | 68 | 5.46 | 0.83 |
| WS 2017/18 | | | | 304 | 4.80 | 1.15 | | | | | | | | | | | | |
| Courses taught according to the ENHANCE concept (total-ENHANCE) | | | | | | | | | | | | | | | | | | |
| SS 2017 | | | | | | | | | | | | | | | | 42 | 5.86 | 0.42 |
| WS 2017/18 | 135 | 5.38 | 0.99 | | | | | | | | | | 181 | 5.60 | 0.81 | 61 | 5.92 | 0.27 |
| SS 2018 | | | | | | | | | | | | | | | | 73 | 5.89 | 0.51 |
| WS 2018/19 | 179 | 5.83 | 0.52 | 223 | 5.17 | 1.12 | 197 | 5.77 | 0.50 | 127 | 5.72 | 0.61 | 106 | 5.88 | 0.38 | 54 | 5.76 | 0.66 |
| SS 2019 | | | | | | | | | | | | | | | | 53 | 5.87 | 0.39 |
| WS 2019/20 | | | | 220 | 4.80 | 1.11 | 200 | 5.82 | 0.55 | 106 | 5.54 | 0.84 | 126 | 5.74 | 0.67 | 31 | 5.48 | 0.62 |

n, sample; M, mean; SD, standard deviation; SS, summer semester; WS, winter semester
